# Postoperative In-Stent Thrombus Formation Following Frozen Elephant Trunk Total Arch Repair

**DOI:** 10.3389/fcvm.2022.921479

**Published:** 2022-06-30

**Authors:** Tim Walter, Tim Berger, Stoyan Kondov, Roman Gottardi, Julia Benk, Bartosz Rylski, Martin Czerny, Maximilian Kreibich

**Affiliations:** Department of Cardiovascular Surgery, Faculty of Medicine, Heart Centre Freiburg University, University of Freiburg, Freiburg, Germany

**Keywords:** frozen elephant trunk (FET), thromboembolism, thrombus, stent graft, postoperative

## Abstract

**Objectives:**

Our aim was to investigate the occurrence and clinical consequence of postoperative in-stent thrombus formation following the frozen elephant trunk (FET) procedure.

**Methods:**

Postoperative computed tomography angiography (CTA) scans of all 304 patients following the FET procedure between 04/2014 and 11/2021 were analysed retrospectively. Thrombus size and location were assessed in multiplanar reconstruction using IMPAX EE (Agfa HealthCare N.V., Morstel, Belgium) software. Patients’ characteristics and clinical outcomes were evaluated between patients with and without thrombus formation.

**Results:**

During the study period, we detected a new postoperative in-stent thrombus in 19 patients (6%). These patients were significantly older (*p* = 0.009), predominantly female (*p* = 0.002) and were more commonly treated for aortic aneurysms (*p* = 0.001). In 15 patients (79%), the thrombi were located in the distal half of the FET stent-graft. Thrombus size was 18.9 mm (first quartile: 12.1; third quartile: 33.2). Distal embolisation occurred in 4 patients (21%) causing one in-hospital death caused by severe visceral ischaemia. Therapeutic anticoagulation was initiated in all patients. Overstenting with a conventional stent-graft placed within the FET stent-graft was the treatment in 2 patients (11%). Outcomes were comparable both groups. Female sex (*p* = 0.005; OR: 4.289) and an aortic aneurysm (*p* = 0.023; OR: 5.198) were identified as significant predictors for thrombus development.

**Conclusion:**

Postoperative new thrombus formation within the FET stent-graft is a new, rare, but clinically highly relevant event. The embolisation of these thrombi can result in dismal postoperative outcomes. More research is therefore required to better identify patients at risk and improve perioperative treatment.

## Introduction

The frozen elephant trunk (FET) procedure has evolved as an effective treatment option in patients suffering from acute and chronic thoracic aortic pathologies including aortic dissection, aortic aneurysms, and penetrating aortic ulcers involving the aortic arch (1–4). However, we have clinically encountered early postoperative thrombus formation and thrombus embolisation within the FET stent-graft component (5). It is therefore our aim to evaluate the occurrence and clinical consequence of postoperative in-stent thrombus formation following the FET procedure.

## Patients and Methods

### Ethics Statement

Our institutional review committee approved this retrospective study, and the need for informed consent was waived (number: 20–1302; approval date: February 4, 2021).

### Patients and Follow-Up Protocol

Between 04/2014 and 11/2021, 304 patients underwent the FET procedure in one aortic centre currently performing over 60 total aortic arch procedures per annum (as of 2021). Computed tomography angiography (CTA) scans were done routinely preoperatively, before discharge, during every follow-up visit, and when clinically warranted. Follow-up was available in all patients after FET implantation.

### Imaging Analysis

All immediate postoperative CTA scans were screened retrospectively. A slice thickness of 3 mm or less was present in all patients. Analysis was performed using IMPAX EE (Agfa HealthCare N.V., Morstel, Belgium). All measurements were taken in multiplanar reconstruction always in plane perpendicular to the manually corrected local aortic centreline.

### Perioperative Approach

Our standardised, integrated surgical management of the FET technique has been reported (6–8). In short, we carry out a full sternotomy and generally cannulate the right axillary artery for arterial inflow for cardiopulmonary bypass. Any concomitant procedures (valve, aortic root, coronary artery) take place while the patients are cooled down a target core body temperature of 25^°^C. We routinely apply cold-blood cardioplegia or the beating-heart technique (using 300 mL of normothermic myocardial perfusion). Bilateral cerebral perfusion is normally used and we liberally perform trilateral antegrade cerebral perfusion (additional cannulation of the left subclavian artery) when needed. For this reason, our preoperative work-up includes a CTA of the supra-aortic vessels including the Circle of Willis. Zone 2 is our standard anastomosis site for FET implantation, and today, we use the short version (100 mm) of the Thoraflex (Terumo Aortic, Inchinnan, United Kingdom) hybrid-graft exclusively. In case of classical aneurysm formation, we oversize the stent-graft component by 10% at the distal landing zone and in case of aortic dissections we avoid oversizing and choose the FET stent-graft size according to institutional standards (1, 9, 10). The diameters of the implanted FET stent-grafts ranged from 22 to 40 mm in this study depending on the preoperative diameter of the aorta in the anticipated landing zone. We do not routinely implant cerebrospinal fluid drainage before surgery.

All patients are routinely transferred to our cardiovascular surgical intensive care unit postoperatively. Intraoperatively, we aim for an activated clotting time longer than 400 s. Intra- and postoperative coagulation is routinely managed *via* rotational thromboelastography (ROTEM) guided in our centre. We commence prophylactic intravenous heparin 6 h postoperatively (500 IU per hour). If therapeutic anticoagulation is required, we raise heparin dosages every 6 h to reach a target partial thromboplastin time of 60–80 s. Anti-platelet therapy (acetylsalicylic acid 100 mg) is routinely commenced on the first postoperative day at noon when no bleeding signs are visible and when only prophylactic heparin is administered.

### Outcome Measures

Data were collected retrospectively relying on our prospectively maintained aortic database. The modified Rankin Scale (mRS) was used to classify the postoperative-stroke severity (11). Consulting neurologists evaluated all the strokes. Postoperative strokes causing no clinical symptoms (mRS 0), no significant disability (mRS 1), or slight disability (mRS 2) were classified as non-disabling postoperative strokes.

### Statistical Analysis

Data are presented as absolute and relative frequency or as median [first quartile, third quartile]. The Student’s *t*-test or the Mann-Whitney-*U*-test was used to compare continuous variables as appropriate. Categorical variables were compared using the Chi-squared test. In case of small group sizes (*n* < 5), Fisher’s Exact test was used. Multivariable logistic regression analyses were performed to investigate the influence of clinically selected variables on postoperative in-stent thrombus formation (selected variables: female sex, age, acute pathology, penetrating aortic ulcer, aneurysm) Statistical analysis was performed using IBM SPSS 21.0 (SPSS Software, IBM Corp., Armonk, NY, United States).

## Results

### Patient and Aortic Characteristics

Within the study period, we identified a new postoperative thrombus in 19 patients (6%). Patient characteristics are summarised in Table 1. Patients developing a thrombus were significantly older (*p* = 0.009), predominantly female (*p* = 0.002) and tended to have a lower incidence of cardiovascular risk factors. Most patients were being treated for a chronic underlying aortic pathologies, but the incidence was comparable between the two groups. Aortic aneurysms were significantly more common (*p* = 0.001), while aortic dissections were significantly less common (*p* = 0.035) in patients with an in-stent thrombus. Of the patients with an in-stent thrombus, one suffered from the coagulation disorder factor-V-Leiden-mutation.

**TABLE 1:** Patient characteristics.

	In-stent thrombi (*n* = 19)	No thrombi (*n* = 285)	*P*-value
Age (years)	74 (66; 77)	67 (58; 73)	0.009
Male	6 (32)	193 (68)	0.002
Current smoker	4 (21)	69 (24)	1.000
Hyperlipidaemia	6 (32)	94 (33)	1.000
Arterial hypertension	14 (74)	242 (85)	0.234
Diabetes mellitus type 2	1 (5)	7 (2)	0.408
History of stroke	3 (16)	36 (13)	0.721
Chronic renal failure	2 (11)	39 (14)	1.000
Chronic obstructive pulmonary disease	5 (26)	25 (9)	0.029
Coronary artery disease	2 (11)	88 (31)	0.070
Connective tissue disease	0 (0)	27 (9)	0.236
Bicuspid aortic valve	0 (0)	14 (5)	1.000
Acute pathology	3 (16)	98 (34)	0.131
Chronic pathology	16 (86)	187 (66)	
Aortic dissection	6 (32)	192 (67)	0.035
Aortic aneurysm	11 (58)	71 (25)	0.001
Penetrating aortic ulcer	2 (11)	22 (8)	0.654

*Values are n (%) or median (first quartile, third quartile).*

### Surgical Details

**FIGURE 1:**
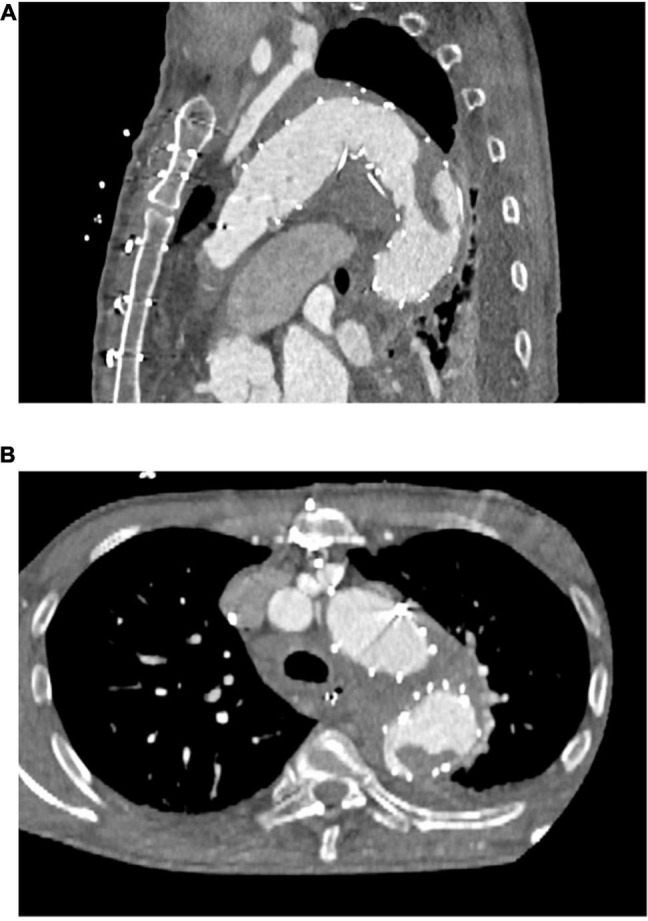
Representative computed tomography angiographic images of a newly detected postoperative thrombus formation within the frozen elephant trunk stent-graft. **(A)** Sagittal plane **(B)** axial plane.

As Table 2 shows, our primary arterial cannulation was the right subclavian artery in most patients. Most concomitant procedures concerned the aortic root or valve. Beating heart technique was applied in almost one third of the patients. There were no statistically significant differences between the two groups. Of the patients with a new in-stent thrombus, one underwent the FET technique after previous thoracic endovascular aortic repair (TEVAR) with no thrombus visible in preoperative CTA scans but a large thrombus visible in the postoperative CTA scan (Figure 1).

**TABLE 2:** Surgical details.

	In-stent thrombi (*n* = 19)	No thrombi (*n* = 285)	*P*-value
**Cannulation**			
Ascending aorta	0 (0)	7 (2)	1.000
Right subclavian artery	19 (100)	269 (94)	0.610
Femoral	1 (5)	7 (2)	0.408
Aortic root replacement	4 (21)	50 (18)	0.698
Aortic valve replacement	5 (26)	37 (13)	0.159
Coronary artery bypass grafting	1 (5)	45 (16)	0.327
Beating heart aortic arch replacement	6 (32)	58 (20)	0.384
Delayed sternum closure	0 (0)	27 (9)	0.236

*Values are n (%) or median (first quartile, third quartile). CABG, coronary artery bypass graft.*

### Thrombus Characteristics

Thrombus characteristics are summarised in Table 3. Median time to postoperative CTA was 7 [5; 11] days and median size of the thrombus was 18.9 [12.1; 33.2] mm. Most thrombi were located in the FET stent-graft’s distal half (*n* = 15, 79%). A new thrombus was detected retrospectively in 9 patients (47%), in this analysis, but they had no clinical relevance during the hospital stay and these thrombi were not visible during follow-up visits (Supplementary Figure 1). Four patients suffered a thrombus embolisation (21%).

**TABLE 3:** Thrombus characteristics.

	*N* = 19
Location in stent-graft*	
Proximal	4 (21)
Distal	15 (79)
Size of thrombus (mm)	18.9 (12.1; 33.2)
Retrospectively detected, no clinical relevance	9 (47)
Embolisation	4 (21)
Coeliac trunk occlusion	3 (16)
Superior mesenteric artery occlusion	2 (11)
Renal artery occlusion	2 (11)
Iliac/femoral artery occlusion	0 (0)

Values are n (%) or median (first quartile, third quartile). *proximal half of the stent-graft or distal half of the stent-graft.

### Thrombus Management

Of the 10 patients diagnosed with a new, relevant postoperative thrombus within the stent-graft, all received therapeutic anticoagulation. TEVAR was performed to over-stent the thrombus in two patients. In their following CTA scans, no thrombus was visible any longer. Oral anticoagulation was the sole treatment of choice in four patients, and their thrombi resolved without any clinical consequence and were no longer visible in follow-up CTA scans.

### Embolisation Management and Outcome

**FIGURE 2:**
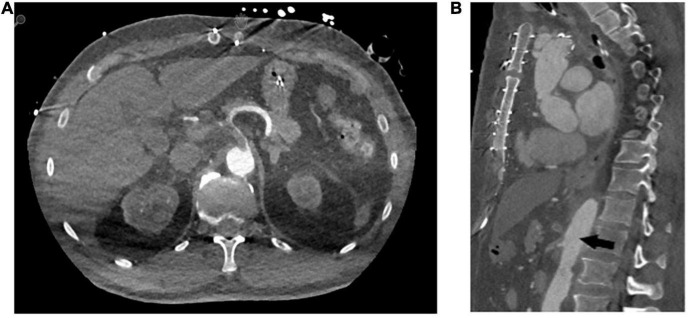
Representative computed tomography angiography images of distal embolisation of the in-stent thrombi to the coeliac trunk **(A)** axial plane **(B)** sagittal plane.

Thrombus embolisation occurred in four patients (21%). In the first, embolisation led to a subtotal occlusion of the coeliac trunk (Figure 2), the superior mesenteric artery and both renal arteries. Because of this patient frail state, we opted for an endovascular approach and were able to re-vascularise the superior mesenteric artery and both renal arteries *via* stent-graft [Advanta stent-grafts (Getinge Deutschland GmbH, Rastatt, Germany)] implantation. It was unfeasible to revascularise the coeliac trunk. Despite all our attempts, this patient developed severe visceral ischaemia and expired in multi-organ failure with circulatory depression. Embolisation in the second patient caused a total occlusion of the left renal artery entailing complete ischaemia of the left kidney. This patient’s medical history included complex interventions and operations of the renal arteries including an iliaco-renal bypass; therapeutic anticoagulation was the treatment of choice. This patient required permanent postoperative dialysis. The third patient’s embolisation caused infarctions in the liver and spleen and a subtotal occlusion of the ileocolic artery. A remaining thrombus was visible in the FET stent-graft that had not resolved in a control CTA scan despite therapeutic anticoagulation. Therefore, a conventional stent-graft (Relay NBS Terumo Aortic, Vascutek Ltd., Inchinnan, United Kingdom) was put in place to over-stent the thrombus. During another control CTA scan, the thrombus had disappeared. In the fourth and last patient, the thrombi embolised inside the coeliac trunk, the splenic, gastroduodenal, and left gastric arteries causing no clinically relevant malperfusion or organ infarction. After initiating therapeutic anticoagulation, the thrombi were no longer visible during the follow-up CTA scan.

All outcome characteristics are summarised in Table 4. There were no statistically significant differences between the two groups.

**TABLE 4:** Postoperative and follow-up outcome.

	In-stent thrombi (*n* = 19)	No thrombi (*n* = 285)	*P*-value
In-hospital mortality	1 (5)	24 (8)	1.000
Stroke	3 (16)	44 (16)	1.000
Non-disabling stroke	1 (5)	13 (5)	1.000
Disabling stroke	2 (11)	31 (12)	
Temporary renal replacement therapy	4 (21)	28 (9)	0.129

*Values are n (%) or median.*

### Logistic Regression Analysis

Female sex (*p* = 0.005; OR: 4.289) and an aortic aneurysm (*p* = 0.023; OR: 5.198) were identified as significant variables in our logistic regression analysis (Table 5).

**TABLE 5:** Logistic regression analysis—in-stent thrombus formation.

Variable	*P*-value	OR	95% CI
Age (years)	0.153	1.043	0.985–1.105
Female sex	0.005	4.289	1.534–11.989
Acute pathology	0.870	1.147	0.222–5.928
Penetrating aortic ulcer	0.203	3.362	0.519–21.759
Aortic aneurysm	0.023	5.198	1.255–21.540

*CI, confidence interval; OR, odds ratio.*

## Discussion

Our study’s most important findings can be summarised as: (i) new postoperative thrombus formation within the FET stent-graft is a clinically significant problem with a relevant incidence; (ii) although embolisations of these thrombi are rare, they potentially cause dismal postoperative outcomes; (iii) additional research evidence is needed to better identify patients at risk and improve perioperative treatment.

This cohort’s chronic health conditions and risk factors were comparable to other patient cohorts undergoing total arch replacement using the FET technique (4, 12, 13). Nevertheless, note that thrombi were diagnosed predominantly in female patients who were significantly older. In fact, female sex was identified as a significant predictor for a new in-stent thrombus development in our logistic regression model. Following conventional TEVAR, female sex is a known risk factor for peripheral stent-thrombosis and stent-graft luminal narrowing (14, 15). Why female patients suffer this higher incidence is unknown, but it may be associated with different aortic flow/compliance in female and/or differences in their coagulation pathways (15). Note that one patient suffered from the factor-V-Leiden-mutation coagulation disorder. Much more research is needed to discover additional risk factors for the occurrence of stent-graft thrombi with a specific focus on gender-related aspects.

In addition, aneurysms were the predominant underlying pathology in this study and an aortic aneurysm was identified as a highly significant variable in our logistic regression model. These patients may have had chronic thrombi formation within the aneurysm sack, but we do not know how these thrombi became dislocated within the FET stent-graft. After all, a crimped stent-graft is introduced into the aorta and intraoperative perfusion in this cohort was always antegrade (axillary cannulation in all patients). The additional cannulation of the femoral artery in one patient who developed a new in-stent thrombus may have caused retrograde introduction of a thrombus to within the stent-graft, as retrograde embolisation has been reported in patients with femoral artery cannulation for cardiopulmonary bypass (16, 17). However, the statistically comparable cannulation approach between the two groups suggests that thrombi developed after FET deployment independent of the cannulation method even though the cause remains unclear.

One major limitation of this study is the lack of perioperative coagulation management data and transfusion requirements. Nevertheless, there is an urgent need for a general awareness of this serious problem to develop within the aortic community, since other large volume aortic centres have also preliminarily reported on this issue (18). Of note, we want to highlight the fact that we identified postoperative in-stent thrombi in 9 patients during our retrospective analysis of the postoperative CTA scans that had been previously undetected and were fortunately clinically irrelevant. Hence, there is an obvious need for in-depth analyses of perioperative coagulation management in larger case series, ideally in a multi-centre analysis of this complication including patients having received both commercially available stent-grafts in Europe. We also want to emphasise that there was no case of delayed sternum closure in patients with a new in-stent thrombus—an indirect marker for no significant bleeding necessitating massive transfusion that may have increased the likelihood of intravascular thrombus formation.

In four patients, the thrombi embolised, causing significant postoperative morbidity and mortality. Our treatment approach comprised therapeutic anticoagulation in all patients. When larger, restiform thrombi are diagnosed in CTA we liberally opt for endovascular over-stenting, thereby paying utmost attention to not dislocate the thrombus through our wire manipulation. By this approach, we implant a conventional stent-graft within the FET stent-graft that provides an excellent proximal landing zone (19) or we implanted smaller stent-grafts into visceral arteries. When thrombus embolisation occurs, we plan our treatment interdisciplinarily with our interventional specialists and our visceral surgeons. In this difficult clinical scenario, any attempt seems plausible to restore visceral perfusion, but outcomes in patients with visceral malperfusion remain dismal (20, 21).

This study shows that postoperative thrombi formation within the FET stent-graft is a new and clinically severe postoperative complication warranting further research and careful postoperative CTA analysis of all patients following the FET procedure. Postoperative surveillance following the FET procedure focussing on individual patient-specific factors is warranted.

### Limitations and Strengths

While we describe a novel complication following the FET procedure, our study is obviously limited by its retrospective nature and the lack of data on the perioperative management of anticoagulation or potential postoperative coagulation disorders such as disseminated intravascular coagulation. In addition, we cannot rule out the development of any thrombus during later stages or in-between two CTA scans. Furthermore, we identified several thrombi in postoperative CTA scans during this retrospective analysis that had no clinical relevance. The possibility remains that buckling or flow effects may also be present in these patients. Nevertheless, our findings of particularly large thrombi clearly highlight this complication’s urgent clinical importance and the need for further research.

## Conclusion

Postoperative new thrombus formation within the FET stent-graft is a new but also clinically a highly relevant event. Female patients and patients with the aortic aneurysms seem to be at higher risk for a new in-stent thrombus formation. Embolisation of these thrombi can cause dismal postoperative outcomes, making additional investigations necessary to better identify patients at risk and improve their perioperative treatment.

## Data Availability Statement

The raw data supporting the conclusions of this article will be made available by the authors, without undue reservation.

## Ethics Statement

Written informed consent was obtained from the individual(s) for the publication of any potentially identifiable images or data included in this article.

## Author Contributions

TW: conceptualisation, formal analysis, methodology, data curation, and writing—original draft. TB: data curation, formal analysis, and writing—review and editing. SK: visualisation and writing—review and editing. RG: data curation, methodology, and writing—review and editing. BR: supervision and writing—review and editing. MC: methodology, project administration, supervision, and writing—review and editing. MK: conceptualisation, methodology, supervision, validation, and writing—review and editing. All authors contributed to the article and approved the submitted version.

## Conflict of Interest

The authors declare that the research was conducted in the absence of any commercial or financial relationships that could be construed as a potential conflict of interest.

## Publisher’s Note

All claims expressed in this article are solely those of the authors and do not necessarily represent those of their affiliated organizations, or those of the publisher, the editors and the reviewers. Any product that may be evaluated in this article, or claim that may be made by its manufacturer, is not guaranteed or endorsed by the publisher.

## Supplementary Material

Supplementary Figure 1 Computed tomography angiographic images of a previously undetected thrombus in the distal end of the stent-graft **(A,C)** axial plane, **(B,D)** sagittal plane.

## Abbreviations

CTA: Computed tomography angiography
FET: Frozen elephant trunk.
