# Supplementary figures and images for: Postoperative In-Stent Thrombus Formation Following Frozen Elephant Trunk Total Arch Repair
Source: Front Cardiovasc Med. 2022 Jun 30;9:921479. doi: 10.3389/fcvm.2022.921479 (PMC9279669; doi:10.3389/fcvm.2022.921479)

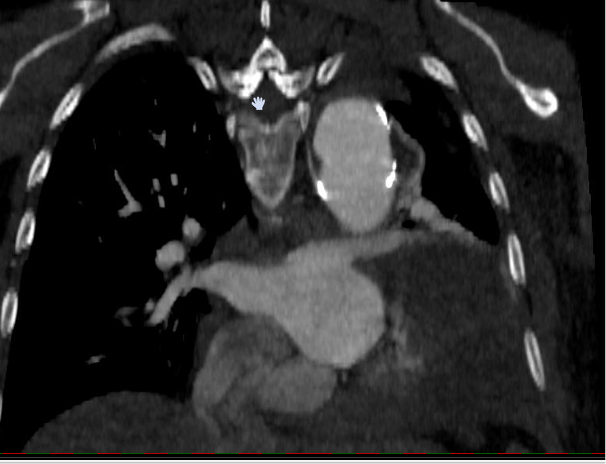

Supplement: Supplementary file 1 [file Image_2.PNG]

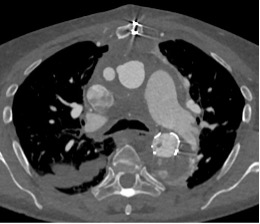

Supplement: Supplementary file 2 [file Image_3.JPEG]

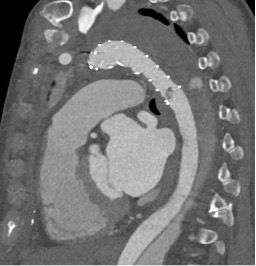

Supplement: Supplementary file 3 [file Image_4.JPEG]

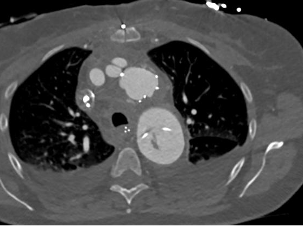

Supplement: Supplementary file 4 [file Image_5.JPEG]

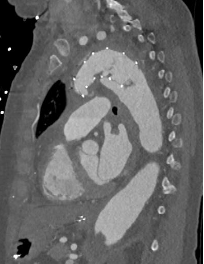

Supplement: Supplementary file 5 [file Image_6.JPEG]

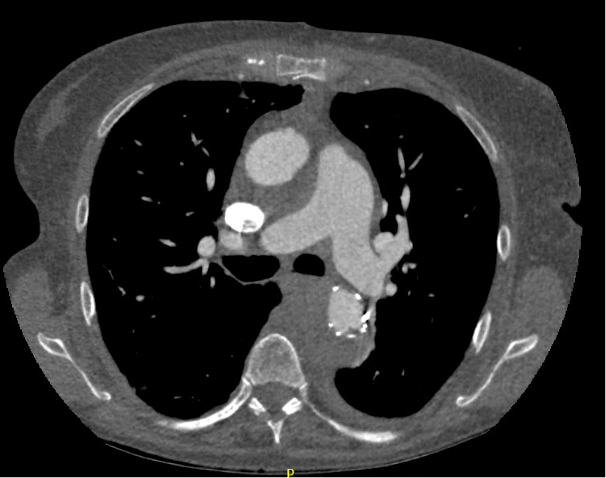

Supplement: Supplementary Figure 1 — Computed tomography angiographic images of a previously undetected thrombus in the distal end of the stent-graft (A,C) axial plane, (B,D) sagittal plane. [file Image_1.PNG]
